# Modeling Skin Conductance Response Time Series during Consecutive Rapid Decision-Making under Concurrent Temporal Pressure and Information Ambiguity

**DOI:** 10.3390/brainsci11091122

**Published:** 2021-08-25

**Authors:** Takahiro Soshi, Mitsue Nagamine, Emiko Fukuda, Ai Takeuchi

**Affiliations:** 1 Department of Community Mental Health and Law, National Institute of Mental Health, National Center of Neurology and Psychiatry, 4-1-1 Ogawa-higashi-cho, Kodaira, Tokyo 187-8553, Japan; soshitakahiro@gmail.com; 2 Institute for Liberal Arts, Tokyo Institute of Technology, 2-12-1 Ookayama, Meguro-ku, Tokyo 152-8550, Japan; 3 Department of Industrial Engineering and Economics, School of Engineering, Tokyo Institute of Technology, 2-12-1 Ookayama, Meguro-ku, Tokyo 152-8552, Japan; fukuda.e.ac@m.titech.ac.jp; 4 Department of Economics, Ritsumeikan University, 1-1-1 Nojihigashi, Kusatsu, Shiga 525-0058, Japan; ai-tak@fc.ritsumei.ac.jp

**Keywords:** decision-making, temporal pressure, information ambiguity, anxiety, skin conductance response, psychophysiological modeling

## Abstract

Emergency situations promote risk-taking behaviors associated with anxiety reactivity. A previous study using the Iowa Gambling Task (IGT) has demonstrated that prespecified state anxiety predicts moderate risk-taking (middle-risk/high-return) after salient penalty events under temporal pressure and information ambiguity. Such moderate risk-taking can be used as a behavioral background in the case of fraud damage. We conducted two psychophysiological experiments using the IGT and used a psychophysiological modeling approach to examine how moderate risk-taking under temporal pressure and information ambiguity is associated with automatic physiological responses, such as a skin conductance response (SCR). The first experiment created template SCR functions under concurrent temporal pressure and information ambiguity. The second experiment produced a convolution model using the SCR functions and fitted the model to the SCR time series recorded under temporal pressure and no temporal pressure, respectively. We also collected the participants’ anxiety profiles before the IGT experiment. The first finding indicated that participants with higher state anxiety scores yielded better model fitting (that is, event-related physiological responses) under temporal pressure. The second finding demonstrated that participants with better model fitting made consecutive Deck A selections under temporal pressure more frequently. In summary, a psychophysiological modeling approach is effective for capturing overlapping SCRs and moderate risk-taking under concurrent temporal pressure and information ambiguity is associated with automatic physiological and emotional reactivity.

## 1. Introduction

Occasionally, people need to make behavioral decisions without sufficient time and information. In such situations, they may decide upon a choice with higher risk, resulting in undesirable consequences. For example, during the ongoing worldwide COVID-19 pandemic, people tend to encounter difficulties in obtaining enough face-to-face information about circumstances. This may lead them to take decisions that may have adverse consequences, such as becoming a fraud victim (e.g., Ministry of Health, Labour, Welfare of Japan 2020 [1] and UK Government 2020 [2]). It is useful to obtain information about how risky decisions are made during an emergency. Since decision-making can be associated with automatic physiological and emotional activity [3,4,5,6,7,8,9], this study aimed to elucidate how such automatic activity is associated with decision-making under emergency conditions, using electrodermal activity and the Iowa Gambling Task (IGT) [3]. The IGT is the most popular decision-making task that simulates risk-taking behavior in real-life circumstances. The task mission is to maximize final winnings by selecting either the disadvantageous decks (Decks A and B) or advantageous decks (Decks C and D) in each trial.

At least two situational factors may affect decision-making behavior, as reported in previous IGT studies: “temporal pressure” and “information ambiguity”. The temporal pressure affects decision-making [10,11,12,13] due to limited resources for online information processing, anticipation of choice outcomes, action execution under monitoring, and outcome evaluation and feedback for subsequent behaviors [14]. Cella et al. (2007) [11], for example, examined how external temporal pressure affects decision-making in the IGT. They applied 2-s and 4-s temporal constraints to card selections in two participant groups and observed that the 2-s constraint induced disadvantageous card deck selections more frequently. Subjectively evoked temporal pressure also promotes disadvantageous card deck selections [12].

Information ambiguity or unresolvable uncertainty [15] concerning circumstances [16,17] is also an important factor in decision-making, because it promotes efforts to solve an uncertainty in an anticipatory manner [14], which may be associated with motivation [18,19,20]. However, a lack of prior information does not necessarily improve risk-taking. Balodis et al. (2006) [21], for example, did not outline an initial selection strategy for avoiding disadvantageous card decks and maximize the rewards in the IGT; such information uncertainty did not improve the selection of disadvantageous card decks even at later trial stages. Similarly, Singh (2013) [22] observed that reward-seeking behavior is distorted without the supportive information of a selection strategy.

One recent IGT study reported for the first time that concurrent temporal pressure and information ambiguity evokes moderate risk-taking (selection of Deck A with frequent middle penalty) after salient penalty events (JPY 125,000 in Deck B) [23]; this risk-taking was also associated with a self-reported state anxiety, which is defined as transient anxious states in response to current emotional events, in contrast to trait anxiety, which is a stable predisposition as a personality trait to frequent experiences of anxiety [24]. Such anxiety-related, moderate risk-taking may be valued as a basic behavioral mechanism for fraud victimization: the state anxiety of people who are vulnerable to future victimization may sensitively change under temporal pressure and ambiguity as a result of a lack of information. Interestingly, although this study observed a moderate correlation between state anxiety and trait anxiety, as in a previous study [25], only state anxiety was positively associated with Deck A selection under temporal pressure and information ambiguity.

Such dissociation between state and trait anxiety may be associated with psychological and neural factors. The psychological factors may be related to automatic emotional reactivity. Higher trait anxiety could be related to (i) anxiety perseveration by attentional bias to threatening information [26,27,28,29] and (ii) reducing the automatic emotional reactivity [30,31] for the avoidance of risk-taking [32]. Under temporal pressure and information ambiguity, people with higher trait anxiety tend to attenuate their automatic emotional reactivity to future events [29,33], which is a process intended to manipulate emotional generation [34]. This is in contrast with the characteristics of state anxiety whereby automatic responses to threatening information could occur under attenuated control, adaptively promoting emotional reactivity [29,35]. Concerning the neural factors, state and trait anxiety may also have different foundations. On the one hand, Saviola et al. [36] reported that state anxiety is associated with posterior cortical areas, such as the precuneus and posterior cingulate cortex within the default mode network (DMN) [37], which play a part in adaptive behavioral changes. On the other hand, trait anxiety is associated with anterior cortical regions, such as the superior and middle prefrontal areas, related to emotional regulation [36]. Another brain imaging study has also reported anxiety-related segregation of the hippocampus [38].

Based on these finding, we hypothesized that moderate risk-taking under temporal pressure and information ambiguity is highly associated with the automatic emotional reactivity evoked by state anxiety. To examine this hypothesis, we used skin conductance activity as an automatic emotion-related physiological change [39,40,41]. This electrodermal activity increases via electric skin conductance in relation to sweat current from the eccrine sweat gland, which is regulated by the sympathetic axis of the hypothalamus-controlled automatic nervous system. Skin conductance activity is also influenced by various subcortical areas, such as the amygdala, thalamus, and cerebellum [4,42,43,44,45], and cortical areas, such as the anterior or posterior cingulate cortex, dorsal or medial prefrontal, supplementary or premotor areas, anterior insula, precuneus [43,44,45,46,47], and the ventral orbitofrontal cortex [5,42,43,44,46,48]. Skin conductance activity can be recorded at the stratum corneum in the epidermis layer and is divided into two types of activities [39]. The skin conductance response (SCR), which is used in the present study, is a phasic physiological response, which is short (several tens of seconds) and modulated by external and internal events. The tonic skin conductance level is a background physiological level that lasts longer. Both these skin conductance measures have been frequently used for investigating emotional sympathetic activity [39,40,41].

Previous IGT studies have used not only SCR but also heart rate variability (HRV) [49,50]. Forte et al. [50], for instance, reported that participants with high-risk avoidance, compared to participants with low-risk avoidance, showed enhanced high-frequency HRV associated with prefrontal inhibitory control. Although the present study simultaneously recorded HRV and SCR during the IGT, this paper focused on SCR to investigate the event-related physiological response time-locked to each trial. Previous IGT studies using SCR have reported two types of event-related responses. On the one hand, anticipatory SCR appears before card selection and reflects on the prediction of the choice outcomes [42,46,48,51,52,53,54,55]. People with damage to their ventral orbitofrontal areas and amygdala attenuate anticipatory SCR [42,48,51]. Appraisal SCR, on the other hand, appears after choice outcomes [42,51,52,53,56,57]. This SCR is interpreted as the successful integration of trial outcomes; people with damage to the amygdala, but not the ventral orbitofrontal areas, attenuate this type of SCR [42,51]. These SCRs tend to be investigated using between-trial or for-analysis intervals, ranging from about 3 to 10 s (e.g., <4 s: Reference [44]; 5 s: References [52,53,56,58,59]; 6 s: References [42,60]; 10 s: References [57,61]). This is because the onset time (latency) and the rise time (duration between the onset and the peak-amplitude times) of SCR tend to range from 1 to 3 s [62,63]. In contrast, we aim to assess SCR over continuous trials under temporal pressure with a 0.5-s feedback duration, and therefore, we face the problem of overlapping SCRs that cannot be decomposed [64,65].

To resolve this analytical problem, we used a model-fitting approach to compare a recorded SCR time series with a linear convolution model of an estimated SCR function (SCRF) [66,67,68,69,70,71]. Bach et al. adopted the linear and time-invariant assumption of SCR. First, the SCR is taken as a variant of a canonical time-invariant response function [67] (cf. see Reference [65] for multiple response functions). Second, the SCR time series continuously produced by multiple events are taken as linear summations of individual SCRs. This approach has the advantage of (i) estimating overlapping SCRs that are hard to decompose and (ii) generalizing a canonical response function to various events and populations by parameter setting [66,67].

This study conducted two IGT experiments. In Experiment 1, we created SCRFs approximated to SCRs recorded under temporal pressure and information ambiguity. In Experiment 2, other participants also performed the IGT under information-ambiguous conditions with and without temporal pressure, separately. Using the SCRFs from Experiment 1, we created a linear convolution model for the recorded SCR time series and calculated fitting coefficients. Subsequently, the fitting coefficients were regressed using state and trait anxiety scores across the participants. Finally, the Deck A selection behaviors were regressed by fitting coefficients. We predicted that model fitting coefficients for the concurrent temporal pressure and information ambiguity condition would be selectively associated with state anxiety scores and Deck A selection, because SCRFs are adjusted for the temporal pressure condition.

## 2. Materials and Methods

### 2.1. Experiment 1

The first IGT experiment aimed to construct an SCRF for each card deck using SCRs recorded under concurrent temporal pressure and information ambiguity. Four SCRFs were applied in the analyses in Experiment 2 to construct an explanatory model for the skin conductance time series measured during the IGT. This experiment was conducted in July 2018.

#### 2.1.1. Participants

Sixteen healthy participants (sex: 9 men and 7 women; age (*M* ± *SD*): 20.6 ± 2.2 years) were recruited through an advertisement at the university of the second and third authors. They were native speakers of Japanese and had normal or corrected-to-normal vision. Participants’ intelligence quotients (IQ (*M* ± *SD*): 109.9 ± 5.7) were assessed using the Japanese version of the National Adult Reading Test [72,73] before the experiment commenced. Before the experiment, they were told that they would be paid (i) no less than JPY 2000 for their participation and (ii) an optional bonus of no more than JPY 1000, depending on their final rewards in the IGT. They provided informed consent per the institutional guidelines, which are in accordance with the Declaration of Helsinki, a statement of ethical principles for medical research involving human participants. This study was approved by the University Ethics Committee of the second and third authors.

#### 2.1.2. Iowa Gambling Task

The IGT asks participants to maximize their final winnings by developing an optimal strategy across all 100 trials [3]. Participants chose one of the four advantageous or disadvantageous card decks in each trial. Decks A and B are disadvantageous decks, which randomly impose high monetary penalties but consistently provide a relatively high reward of JPY 10,000. Deck A is referred to as a “middle-risk deck” with a high return but a frequent high loss of JPY 15,000–35,000. Deck B, which is a “high-risk deck,” carries the rare maximum penalty of JPY 125,000 in each set of 10 cards. Decks C and D are advantageous decks, imposing smaller penalties but also providing lower rewards of just JPY 5000. Monetary losses frequently occur in Deck C, ranging from JPY 2500–7500. In Deck D, a relatively high penalty of JPY 25,000 occurs infrequently in each set of 10 cards. The present study used a version of the IGT program implemented in Cognitive Experiments V v1 (Neurobehavioral Systems Inc., Berkeley, CA, USA) modified for the Japanese language.

#### 2.1.3. Psychological Assessment

Participants’ state and trait anxiety were self-assessed by the Spielberger State–Trait Anxiety Inventory (STAI) [74,75,76]. This scale assesses the levels of state anxiety (STAI-S) and trait anxiety (STAI-T) through 40 items.

#### 2.1.4. Procedure

The participants came to a quiet experimental room to participate in the experiment. They filled in the anxiety scale questionnaire 1 h before the IGT. The anxiety scores were compared with those in Experiment 2 to confirm the homogeneity of baseline anxiety profiles between the participants in Experiments 1 and 2. After answering the anxiety scale questionnaire, the participants performed the IGT. As was argued in a previous study [77], the manner of task instruction affects participants’ strategies. In contrast to the original instructions [6], we enhanced the ambiguity of the task-related information by not explaining the deck types or offering a guiding strategy. After the practice session for familiarization with the response pad (RB-740, Cedrus Corp., San Pedro, CA, USA), the participants carried out one session of the IGT, which was projected on a 19-inch PC monitor (Dell UltraSharp 1909W, DELL Inc., Round Rock, TX, USA) placed 0.65 m ahead of them. They were instructed to select a card deck as soon as possible after viewing the deck selection display, thus imposing temporal pressure. The alarm (“Choose quickly!”) flashed immediately below the center of the four decks until the participants selected a deck. The feedback display appeared for 500 ms, and the next trial started 10 s after the previous trial. This between-trial interval may still be too short to extract SCR for each trial with full recovery to the baseline. Nevertheless, this interval was used because intervals longer than 10 s may result in low-pressure performance similar to self-paced selection and, thus, are not suitable for creating SCRFs that reflect genuine emotional reactivity under temporal pressure.

#### 2.1.5. Data Acquisition

Skin conductance data were recorded during the IGT from the tips of the left index and middle fingers using 6-mm Ag/AgCl nonpolarizable electrodes filled with 0.05-mol NaCl electrode gel (GEL101, BIOPAC System. Inc., Goleta, CA, USA). The electrodes were mounted on a skin conductance transducer (TSD203) connected to the recording unit (EDA100C), which was connected to an analog-to-digital converter (MP150). The recording unit applied a constant voltage (0.5 V) to the transducer. The data were recorded using data acquisition software (AcqKnowledge ver. 5.0.1) with a sampling rate of 1000 Hz, which was amplified with a band-pass frequency from DC to 10 Hz with a gain of 2 μS/V.

#### 2.1.6. Data Analysis

##### Behavioral Performance

Previous studies observed lower risk-avoidance behavior under information ambiguity conditions [21,22], and adding temporal pressure to information ambiguity also induced this tendency [23]. Following these studies, our analysis of Experiment 1 also employed a net score as the index of risk-avoidance preference. This score was calculated for each block (20 trials) by subtracting the number of selected disadvantageous decks (Decks A and B) from that of the selected advantageous decks (Decks C and D). An increase in net scores in later blocks indicated the development of risk-avoidance behavior. The net scores were compared using one-way repeated measures analysis of variance (ANOVA) with the block factor. When a significant block effect appeared, multiple comparisons were conducted with Bonferroni correction. The significance level of α was set to *p* < 0.05. The statistical test was conducted using SPSS version 25.0 (IBM Corp., Armonk, NY, USA).

##### Skin Conductance Response

Skin conductance data for each participant were initially filtered with a high-pass frequency of >0.033 Hz. In total, 100 trial epochs were extracted from “1 s before” to “10 s after” trial onset and were baseline-corrected with average amplitudes during the baseline interval (1-s duration). The segmented epochs were divided into four deck types according to the participant’s deck selection. Epochs for each deck type (Mean epochs: Deck A = 19, Deck B = 36, Deck C = 21, and Deck D = 24) were introduced into a principal component analysis, and dominant components explaining over 80% of the total epochs were obtained for reconstructing SCR waveforms. Finally, the reconstructed SCRs were averaged for each deck type and were baseline-corrected again to modify the baseline shifts.

To produce SCRFs, the individually averaged SCRs were initially scaled into Z-scores across the four deck types for each participant. The scaled SCRs were averaged separately for each deck type across the participants. The total averaged SCR for each deck type was fitted using a canonical response function modeled as the linear summation of a Gaussian function with a Gamma distribution and an exponential function, as derived from the influential study in Reference [67]: SCRF(t) = N(t)⊗(E_1_(t) + E_2_(t)), where ⊗ is the linear convolution operator, $N\left(t\right)=\frac{e^{\frac{-{\left(t-\tau \right)}^{2}}{2\sigma^{2}}}}{\sqrt{2\pi \sigma }},$ and $E_{1}\left(t\right)=e^{-\lambda_{1}t};{\;E}_{2}\left(t\right)=e^{-\lambda_{2}t}.$ N(t) is a Gaussian function, which includes parameters of τ as the center of the distribution for determining the peak latency of the SCRF and standard deviation σ for determining the inclination (that is, the rise time of the SCR) of the function. E(t) is a decay function, in which the symbols λ_1_ and λ_2_ represent coefficients that determine the full time to recover to the baseline. Based on the original SCR for Deck B, comprising maximum penalty events, the initial parameters were manually set to τ = 3.870, σ = 1.300, λ_1_ = 0.262, and λ_2_ = 3.513 for the four deck types. The length of the time series (t) of an SCRF was 30 s after the trial started. Subsequently, an optimal value for approximation was determined for each parameter, with the other three parameters set in the order of τ, σ, λ_1_, and λ_2_, based on the criterion that the fitness between an observed SCR and an SCRF should possess the highest determination coefficient (*R*^2^). The estimated values of the four parameters ranged from 0 to 10, and the resolution for parameter estimation was equal to the division of the range by 10,000 (0.0001). The approximation continued when the gain improvement of the determination coefficient for at least one deck type was ≥0.0001. We also produced temporal-derivative SCRFs by changing the peak latency or the τ parameter, ranging from no delay to a 10-s delay, because the setting of the intertrial interval in Experiment 1 was different from that of Experiment 2; therefore, derivative SCRFs would be more suitable for explaining the observed SCR time series in Experiment 2. Consequently, 11 SCRFs were obtained for each deck. Analyses were conducted using MATLAB ver.2019b (MathWorks Inc., Natick, MA, USA) and depended on the function “scr_bf_crf.m” for producing a canonical response function, implemented in the open software SCRalyze b2.1.8 (scralyze.sourceforge.net, accessed on 30 June 2021) [68], which has been updated to the PsPM [71].

### 2.2. Experiment 2

The second experiment aimed to construct an explanatory model for each participant’s skin conductance time series during the IGT, using the SCRFs from Experiment 1. We also aimed to examine how model fitting as an index of event-related, automatic physiological reactivity was associated with prespecified anxiety scores and Deck A selection as moderate risk-taking. Behavioral results have already been extensively reported in previous studies [23]; therefore, this study mainly analyzed the SCR data and moderate risk-taking behaviors (Deck A selection) in order to examine the relationship between them. Experiment 2 was conducted prior to Experiment 1 between March and April 2018.

#### 2.2.1. Participants

We enrolled 33 participants (sex: 19 men, 14 women; age: 21.9 ± 2.3 years; IQ: 111.2 ± 5.5) who did not participate in Experiment 1 by means of an advertisement at the University of the second and third authors. The characteristics of the participants were not significantly different from those of the participants in Experiment 1 (sex ratio: χ^2^ = 0.008, *p* = 0.930; age: *t* = 1.833, *p* = 0.073; IQ: *t* = 0.768, *p* = 0.446). The participants were Japanese native speakers and had normal or corrected-to-normal visual acuity. They received JPY 2000 for participation and an optional bonus of no more than JPY 1000 depending on their final gains. Written informed consent was obtained from the participants according to the institutional guidelines before conducting the experiment. This study was approved by the University Ethics Committee of the second and third authors.

#### 2.2.2. Procedure

We used the same software program for the IGT and almost the same experimental procedure as in Experiment 1, while Experiment 2 included a no temporal pressure condition, as well as the temporal pressure condition. Participants were not provided with information about the total number of trials, deck types, or a preferable strategy for maximizing their final reward. Under the no temporal pressure condition, participants were provided with enough time to select one of the four card decks at their own pace. The feedback display showed the current reward and penalty, as well as the total payoff for 2500 ms. The temporal pressure condition in Experiment 2 did not use the between-trial interval of 10 s; the feedback display transiently appeared for 500 ms, and the next trial started imminently. The task order was counterbalanced across the participants. Participants’ self-reported anxiety profiles were also collected about 1 h before the IGT via the STAI.

#### 2.2.3. Data Acquisition and Analysis

The skin conductance time series during the 100 trials were recorded with the same acquisition settings as were used in Experiment 1. Data for each participant were first separated into different time series for the temporal pressure and the no temporal pressure conditions, respectively. These data covered the interval from 30 s before the first trial to 10 s after the last trial. Data for both conditions were filtered with a high-pass frequency of >0.033 Hz. Information on the trial-onset times and types of selected decks was extracted from log files produced by the software program. A linear convolution model for each participant’s SCR time series was produced by convolving an SCRF for each deck type at the start of each trial. The SCRFs from Experiment 1 included the original non-delay and the 10 temporal-derivative SCRFs [70]; as such, 11 convolution models were constructed for each participant. As the duration of the IGT without temporal pressure was longer than that of the IGT under temporal pressure, comparing model-fitting properties between the two conditions may not be methodologically precise. Therefore, a longer skin conductance time series for the no temporal pressure condition was down-sampled to approach the length of the temporal pressure condition for each participant. Fitting between the recorded SCR time series and the corresponding model for each participant was represented by the beta coefficient (β) employed in a univariate regression analysis; a fitting coefficient represents the extent to which participants yield an event-related SCR. Model-fitting coefficients were regressed by STAI-S and STAI-T scores and the controlling variables of age, sex, IQ, and task orders across participants under each condition. As the previous study observed that temporal pressure evoked a moderate risk-taking behavior immediately after maximum penalty events that was associated with state anxiety scores [23], the present study also regressed moderate risk-taking behaviors via model fitting coefficients (6-s delay model) and the controlling variables (age, sex, IQ, and task order) in a multivariate regression analysis. As argued in the introduction, it is difficult to extract a single SCR waveform for each trial in Experiment 2 because of the overlapping SCRs with a short between-trial interval; therefore, in accordance with the modeling of the overall SCR time series, we used the overall behavior of Deck A selection (proportions (%) of the total selections, including successive selections) as an index for moderate risk-taking. Analyses were conducted using SPSS and MATLAB ver.2019b.

## 3. Results

### 3.1. Experiment 1

#### 3.1.1. Behavioral Performance

**Figure 1:**
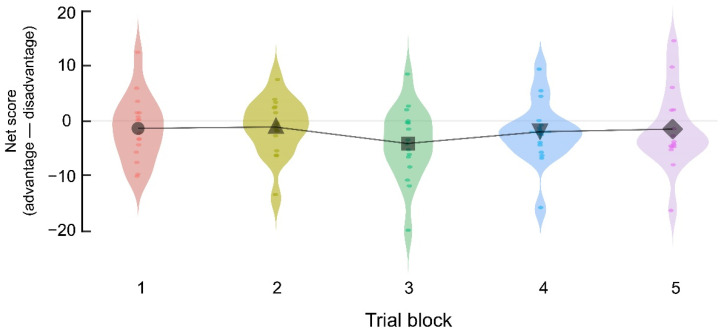
Comparisons of the net scores (advantage (Decks C + D)—disadvantage (Decks A + B)) between the five trial blocks, each of which includes 20 trials in Experiment 1 (*n* = 16). The net scores did not significantly change as the IGT progressed. Each dot represents the net score of each participant in each trial block. Black markers in the five blocks indicate mean net scores.

The behavioral outcomes of the final rewards, proportions of deck selection, and the net scores of the five trial blocks are summarized in Table 1. As confirmed in Figure 1, the net scores showed no significant differences between the blocks (*F*(4, 60) = 1.128, *p* = 0.352, partially (_p_) η^2^ = 0.070); that is, the temporal pressure and enhanced information ambiguity attenuated the development of risk-avoidance preference (gradual increase in net scores). This result is consistent with previous finding [23], which observed lower risk-avoidance under temporal pressure and information ambiguity compared to previous observations of IGT.

#### 3.1.2. Skin Conductance Response

**Figure 2:**
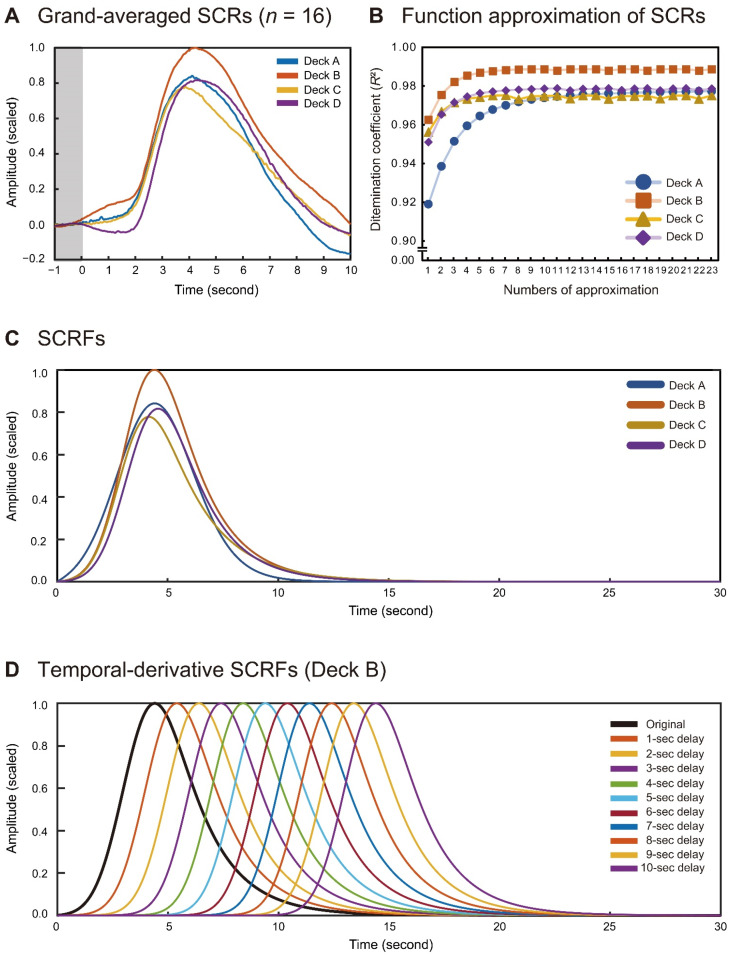
Comparisons of total averaged SCRs for the four card decks in Experiment 1 (*n* = 16) (**A**). Transition of determination coefficients (*R*^2^) during the recursive function approximation of the original SCRs (**B**). SCRFs obtained for the four card decks (**C**) and temporal-derivative SCRFs exemplified for Deck B (**D**).

The individually averaged SCRs were initially scaled to Z-scores for each participant and then averaged across all participants. The grand-averaged waveforms were scaled with the maximal value across the four card decks (i.e., the peak amplitude of Deck B) (Figure 2A). The peak latencies were 4116, 4222, 3861, and 4333 ms for the four deck types, respectively. The half recovery times from the peak latencies were 2296, 2507, 2716, and 2498 ms for the four deck types, respectively. Relative to the maximal amplitude of Deck B, the peak amplitudes of Decks A, C, and D were 0.842, 0.778, and 0.812, respectively.

The grand-averaged SCRs were used to create the SCRFs for each card deck. Function approximation was performed 23 times until no card deck yielded a gain improvement of >0.0001 in the determination coefficient (*R*^2^) (Figure 2B). The four parameters τ, σ, λ_1_, and λ_2_ were determined for the best-fitting SCRFs, as summarized in Table 2, and the SCRFs are plotted in Figure 2C. The peak latencies of the SCRFs were 4412 ms for Deck A, 4402 ms for Deck B, 4150 ms for Deck C, and 4569 ms for Deck D. The half-recovery time durations were 2098 ms, 2123 ms, 2205 ms, and 2105 ms, respectively, in Decks A–D. Relative to the peak amplitude of the SCRF for Deck B, those for Decks A, C, and D were 0.842, 0.778, and 0.817, respectively. The original SCRFs for each card deck were extended to 10 temporal-derivative SCRFs, until a 10-s delay at a 1-s step, and 11 SCRFs, we’re finally introduced into the analyses in Experiment 2 (Figure 2D for Deck B).

### 3.2. Experiment 2

The behavioral outcomes of final rewards, the proportions of deck selection, and the net scores of all trial blocks are summarized in Table 1. Under temporal pressure coupled with information ambiguity, the participants in Experiments 1 and 2 displayed no significant differences in these outcomes, as confirmed by one-way ANOVA for the final reward (group: *F*(1, 47) = 0.128, *p* = 0.722, _p_η^2^ = 0.003), two-way ANOVA for deck selection (group: *F*(1, 47) = 0.480, *p* = 0.492, _p_η^2^ = 0.010; group × deck: *F*(3, 141) = 0.790, *p* = 0.466, _p_η^2^ = 0.017, ε = 0.728), and two-way ANOVA for the net score (group: *F*(1, 47) = 1.153, *p* = 0.288, _p_η^2^ = 0.024; group × block: *F*(4, 188) = 0.836, *p* = 0.489, _p_η^2^ = 0.017, ε = 0.861). The state and trait anxiety scores at the baseline (before the IGT) were also not significantly different between Experiments 1 and 2 (STAI-S: Experiments 1 and 2 = 42.6 ± 7.8, 40.8 ± 6.6; *t* = 0.866, *p* = 0.391; STAI-T: Experiments 1 and 2 = 43.2 ± 9.2, 43.3 ± 7.3; *t* = 0.035, *p* = 0.972). The average scores across all participants (*n* = 49) were 41.4 ± 7.0 for STAI-S and 43.2 ± 7.9 for STAI-T, respectively. The scores of the present participants are approximately five points lower than the Japanese normative data of graduate students (*n* = 333; STAI-S: 46.8 ± 8.5; STAI-T: 48.3 ± 8.3) [74], while the normative data were collected about 30 years ago and may not be applicable to our younger participants.

**Figure 3:**
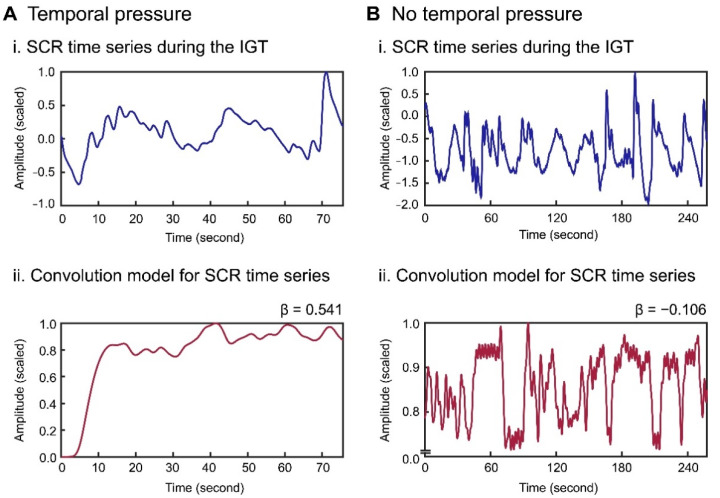
SCR time series recorded during the IGT (**i**) and the corresponding linear convolution model (6-s delay model) using SCRFs (**ii**) for one participant under the temporal pressure conditions (**A**) and the no temporal pressure conditions (**B**).

The skin conductance time series for each participant was initially high-pass-filtered to remove slow baseline drift for both conditions (Figure 3(Ai) for temporal pressure and Figure 3(Bi) for no temporal pressure). A fitting model for the filtered skin conductance time series was created for each participant by linearly convolving each SCRF (Figure 2C) deposited at the onset of each trial (Figure 3(Aii,Bii)). The SCRFs for each participant included the original non-delay function and the 10 derivative functions (Figure 2D), and 11 convolution models were created for fitting the models to the recorded SCR time series. The 10 initial trials comprised an exploratory phase for developing a strategy of deck selection [48,53]. Accordingly, these early trials were discarded from the model fitting analysis.

**Figure 4:**
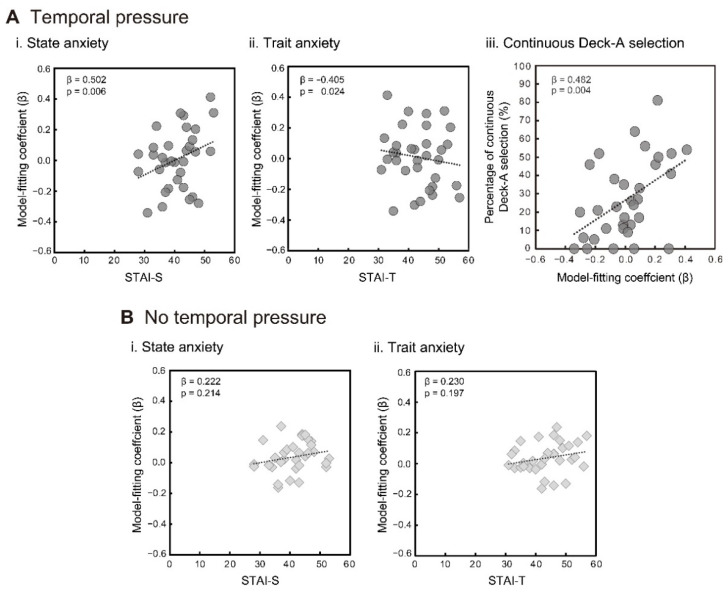
Relations between the participants’ anxiety scores (STAI-S and STAI-T) and their model fitting coefficients (β) for the temporal pressure condition (**Ai**,**Aii**) and the no temporal pressure condition (**Bi**,**Bii**) (*n* = 33). Relation between continuous Deck A selection and model fitting coefficients for the temporal pressure condition (**Aiii**). Beta coefficients were calculated via multiple regression analyses, while the original data were plotted in each graph.

The results of the regression analyses for the two temporal conditions are shown in Table 3 and Table 4. Under the temporal pressure condition, participants’ prespecified STAI-S and/or STAI-T effectively predicted model fitting variations with a peak–delay range of 5 to 8 s. Generally, higher STAI-S scores were associated with better model fitting (Figure 4(Ai)), and inversely, higher STAI-T scores were associated with lower model fitting (Figure 4(Aii)). With regard to the no temporal pressure condition, STAI-S and STAI-T did not effectively explain any model fitting variation (Figure 4(Bi,Bii) for the corresponding 6-s delay model). These results indicate that when participants self-reported higher state anxiety; automatic physiological responses, which were synchronized to IGT trials and modeled by SCRFs, were more sensitively evoked under temporal pressure.

Finally, we predicted the Deck A selection behaviors using the fitting coefficients for the best 6-s delay model. The proportions of continuous Deck A selection under temporal pressure increased as the participants’ model fitting became higher (adjusted *R*^2^ = 0.208, *F* = 9.396, *p* = 0.004; fitting coefficient: β = 0.482, *t* = 3.065, *p* = 0.004, VIF = 1.000) (Figure 4(Aiii)). The no temporal pressure condition did not yield a significant regression model (best model: adjusted *R*^2^ = 0.018, *F* = 1.587, *p* = 0.217). On the contrary, based on the proportions of total Deck A selection, including successive selections as a subset, neither condition yielded a significant association with the fitting coefficients for the 6-s delay model (temporal pressure: β = 0.249, *t* = 1.582, *p* = 0.124; no temporal pressure: β = −0.046, *t* = 0.274, *p* = 0.786), while both analyses obtained the same significant regression model, including the controlling variable of sex (adjusted *R*^2^ = 0.172, *F* = 7.637, *p* = 0.010; sex: β = −0.445, *t* = 2.764, *p* = 0.010, VIF = 1.000).

## 4. Discussion

The present study examined the emotion-related physiological correlates of moderate risk-taking during IGT under conditions of concurrent temporal pressure and information ambiguity. To solve the problem of overlapping SCRs during successive rapid trials with short between-trial intervals [64,65], we used a psychophysiological modeling approach [66,67,68,69,70,71]: the SCRFs were initially produced based on original SCRs (Experiment 1), and a convolution model using the SCRFs for each participant was fitted to an SCR time series recorded during the IGT (Experiment 2). A model fitting coefficient (β) was used as an index of event-related physiological reactivity, which was time-locked to each IGT trial. The participants’ model fitting coefficients for the temporal pressure condition were positively associated with the state anxiety scores. They were also positively associated with continuous Deck A selection under the temporal pressure conditions. These findings support our prediction that moderate risk-taking behavior under temporal pressure and information ambiguity is associated with automatic physiological reactivity, which is positively related to self-assessed state anxiety. In the following, we will first argue for a psychological mechanism of moderate risk-taking under temporal pressure; second, about the psychophysiological nature of SCR modeled by SCRF, and third, about the effectiveness of the psychophysiological modeling approach.

First, our main findings indicate that moderate risk-taking under temporal pressure and information ambiguity is positively associated with anxiety-related, automatic physiological responses. A previous study argued that there are two possible psychological mechanisms for the anxiety-related moderate risk-taking that was observed after maximum-penalty events under conditions of temporal pressure and information ambiguity [23]. The first mechanism is emotional dysregulation—decision-making in the IGT is related to the two stages of processing somatic states [53]. The primary inducer (the first stage) is an external factor, such as an event that automatically evokes a somatic state. The secondary inducer (the second stage) is associated with the internal processing of emotional experiences. Under the temporal pressure condition, participants with higher states of anxiety may not regulate their emotional reactivity and continuously take moderate risks without sufficient processing resources.

The second mechanism is compensation under control. Decision-making during the IGT is associated with three stages of processing: anticipation, action execution under control, and outcome evaluation for feedback [14]. Throughout these recursive processing stages, participants under no temporal pressure generally show less disadvantaged deck selection and feedback monitoring [78,79]. On the other hand, participants with higher state anxiety, when subjected to temporal pressure and information ambiguity, may intentionally choose the moderate-risk deck, particularly after salient penalty events [23], showing an attempt at attentional control to recover the high monetary losses. Although the participants in Experiment 2 self-reported after the IGT that they tended to construct their own selection strategy, the present results seem to suggest the first mechanism: automatic emotional reactivity represented by a model-fitting coefficient is associated with moderate risk-taking under temporal pressure. A previous study reported that participants self-reporting higher state anxiety more frequently selected Deck A after maximum penalty events (JPY 125,000 on Deck B) when subjected to temporal pressure and information ambiguity [23]. Temporal pressure may deprive participants of processing resources for anticipation, action execution, and feedback, as well as may increase their automatic emotional reactivity or SCR, consequently leading to continuous moderate risk-taking.

This interpretation of moderate risk-taking as a product of automatic emotional dysregulation is also supported by the inverse proportional association between model fitting strength and trait anxiety scores under temporal pressure. In Experiment 2, participants self-reporting higher trait anxiety tended to show reduced model fitting strength. This dissociation between state and trait anxiety is likely related to functional and neural differences. On one hand, as argued in the introductory section, trait anxiety is related to (i) attentional bias to anxiety-evoking information [26,27,28,29] and (ii) attenuating emotional reactivity (e.g., SCR) [39,40,41] under control [29,30,31,33]. State anxiety, on the other hand, increases the automatic emotional reactivity to anxiety-evoking information [29,35]. Recent neural findings also support the distinction between state and trait anxiety [36]—trait anxiety is associated with the increased functional connectivity of the superior and middle prefrontal areas within the DMN for emotional regulation. Enhanced activation in the anterior cingulate cortex during a decision-making task has also been reported for higher trait anxiety [80]. State anxiety is associated with increased local functional connections within the posterior regions of the DMN, such as the precuneus and posterior cingulate cortex. A recent physiological study has provided additional evidence that, as the power of high-frequency HRV for prefrontal inhibition increases, cognitive/motor performance is improved in people with higher trait anxiety [81]. To summarize these findings, on the one hand, participants reporting higher trait anxiety may activate a prefrontal inhibition system to suppress automatic emotional reactivity during the IGT and produce a more negative association between model fitting coefficients and trait anxiety scores. On the other hand, participants with higher state anxiety scores may activate the posterior parietal regions independently of the connected rostral and medial prefrontal areas [82]. As maintaining the frontoparietal connection within the DMN may be important for successfully executing cognitive tasks [83], disconnected functioning in the posterior regions may reduce prefrontal inhibition and evoke automatic emotional reactivity during the IGT when under temporal pressure.

Second, we should also discuss the psychophysiological nature of SCR as evoked by temporal pressure. In the present study, the convolution models with peak delays from 5 to 8 s in the temporal pressure condition were explained by self-reported state anxiety (Table 3). Considering that the mean RT for card deck selection was about 1 s, and the feedback duration was 0.5 s, onsets of SCRs under temporal pressure may begin after the disappearance of the feedback display, although the peak latency of, for example, the 5-s delay SCRF in Figure 3B (9 s) is still greater than the estimated peak latency (7.5 s = 1 s (mean RT) + 0.5 s (feedback duration) + 3 s (assumed onset time of SCR) + 3 s (assumed rise time of SCR)). As stated in the introduction, there are two types of SCRs: first, anticipatory SCR, which appears several seconds before deck selection [42,46,48,51,52,53,54,55], and second, appraisal SCR, which appears after receiving choice outcomes [42,51,52,53,56,57]. Considering the estimated onset time and rise time of the SCRs under the temporal pressure condition, the SCR time series explained by the delay models is comprised mainly of appraisal SCRs, which reflect automatic responses after feedback information.

Finally, we should address the method of analysis for continuous SCR time series. The present results indicate that psychophysiological modeling is useful for examining overlapping SCRs during successive rapid decision-making trials. As the mean response time for card selections under temporal pressure was approximately 1 s (713 ± 631 ms), and the feedback duration was also short (500 ms) in Experiment 2, the intervals between adjacent trials were approximately 2 s. This temporal setting is shorter than the estimated interval covering the latency and rise time of an ordinary SCR (approximately 6 s) [62,63], and is not sufficient for the decomposition of independent SCRs with full recovery to the baseline. Therefore, we used a psychophysiological modeling approach that statistically adjusts the parameters of a canonical response function, which formalizes the association between physiological outputs (SCR) and concealed neuropsychological inputs [67,71] based on the outcomes of Experiment 1. We also assumed nonuniformity in the SCRFs for the four types of deck selections. The morphology of SCRs, such as peak latencies, peak amplitudes, and recovery times, would vary across these deck selections, because the four decks were predicted to modulate the intensity of emotional inputs for a response function, according to the different levels of monetary gain and loss. In this study, SCRFs were highly approximated to the recorded SCRs (*R*^2^ scores in Table 2) and actually showed variations in morphology. Furthermore, linear convolution models that summarized these SCRFs effectively captured between-participant variation in event-related physiological reactivity under the temporal pressure condition. Such modeling by the tailor-made SCRFs may offer more than a functional approximation of the original SCRs recorded specifically in our experimental setting. The modeling approach for generating SCR comprises a principal mechanism (that is, a canonical response function between inputs and outputs) and parametric modulation; as argued previously [67], a canonical response function should be parametrized for the peak latency (Gaussian function) and recovery time (decay exponential function) of physiological responses, which could be applied specifically to our participants and experimental settings. This suggests that a uniform response function can be generalized across various populations and experimental tasks to formalize a sequential relationship between neuropsychological inputs and physiological outputs.

## 5. Conclusions

The present study seems to verify that moderate risk-taking under temporal pressure and information ambiguity is associated with anxiety-related physiological reactivity. Although salient risk-taking behavior tends to attract attention in negative social contexts, moderate risk-taking can be amplified under emergency conditions, consequently increasing potential damages. As argued previously [84], the present findings imply multiple strategies for building conditions that protect individuals from fraud victimization. For example, as recruited in this study, younger generations tend to encounter novel types of fraud more frequently, such as subscription and “information for sale (how-to gain high income)” via SNS and the internet in, for example, our country [85]. Social protection may be required to preclude intentional ambiguity established by shortages or excesses of information. Personal protection may also be necessary to prevent subjectively evoked emergencies and higher-state anxiety. Such a multidimensional protection may help in reducing anxiety-related moderate risk-taking under temporal pressure and information ambiguity. We should promote future studies of decision-making under emergent conditions in additional younger participants and other generations, such as older people.

## Figures and Tables

**Table 1:** Behavioral results of the IGT in Experiments 1 and 2.

Behavioral Outcome	Experiment 1 (*n* = 16)		Experiment 2 (*n* = 33)			
	Temporal Pressure		Temporal Pressure		No Temporal Pressure	
	*M*	*SD*	*M*	*SD*	*M*	*SD*
Final reward (JPY)	−50,781	64,276	−38,485	129,293	−35,000	94,765
Total deck selection (%)						
Deck A	18.8	5.7	19.3	8.3	19.8	6.4
Deck B	36.3	13.2	30.7	14.5	34.3	15.3
Deck C	20.8	10.4	23.7	17.8	20.3	10.6
Deck D	24.1	6.6	26.4	12.0	25.6	11.8
Net score ([C + D] − [A + B])						
Block 1	−1.4	5.9	−1.6	9.0	−3.2	5.2
Block 2	−1.1	5.2	0.8	9.6	−0.8	5.3
Block 3	−4.1	6.6	0.4	8.3	−1.0	7.7
Block 4	−2.0	5.8	0.7	9.9	−2.2	10.0
Block 5	−1.5	7.1	−0.2	9.4	−1.0	9.7

**Table 2:** Four parameters showing the best SCRFs for the four card decks.

Deck Type	*R* ^2^	Parameters for SCRF			
		Function 1		Function 2	
		τ	σ	λ_1_	λ_2_
Disadvantage					
Deck A	0.9772	3.6340	1.5420	0.0017	0.9590
Deck B	0.9887	3.3840	1.1370	0.0179	1.8170
Advantage					
Deck C	0.9750	3.0990	1.0510	0.0199	2.1030
Deck D	0.9786	3.5620	1.1520	0.0167	1.7570

SCRF: skin conductance response function; Function 1: Gaussian function; Function 2: decay exponential function.

**Table 3:** Results from multiple regression analyses for the temporal pressure condition (*n* = 33) concerning the associations between participants’ model fitting coefficients and self-reported anxiety scores.

Model Type	Adjusted *R*^2^	*F*-Value	*p*-Value	STAI-S			STAI-T		
				β	*t*-Value	*p*-Value	β	*t*-Value	*p*-Value
Original	0.076	3.634	0.066	0.037	0.213	0.832	−0.063	0.365	0.717
1-s delay	-	-	-	-	-	-	-	-	-
2-s delay	-	-	-	-	-	-	-	-	-
3-s delay	-	-	-	-	-	-	-	-	-
4-s delay	-	-	-	-	-	-	-	-	-
5-s delay	0.199 *	3.650	0.024	0.479 *	2.689	0.012	−0.371 *	2.077	0.047
6-s delay	0.279 **	5.121	0.006	0.502 **	2.969	0.006	−0.405 *	2.384	0.024
7-s delay	0.253 **	4.617	0.009	0.484 **	2.815	0.009	−0.370 *	2.140	0.041
8-s delay	0.195 *	3.580	0.026	0.443 *	2.482	0.019	−0.310	1.728	0.095
9-s delay	0.067	3.299	0.079	0.278	1.672	0.105	−0.064	0.369	0.715
10-s delay	0.060	3.038	0.091	0.264	1.576	0.126	−0.025	0.145	0.886

The coefficients (β) of STAI-S and STAI-T in nonsignificant models (*p*-values for regression models: >0.05) are values if included in regression models. STAI-S: Spielberger State–Trait Anxiety Inventory (State); STAI-T: Spielberger State–Trait Anxiety Inventory (Trait); *: *p* < 0.05 and **: *p* < 0.01. All variables were excluded from the regression model, and the adjusted *R*^2^ was zero.

**Table 4:** Results from multiple regression analyses for the no temporal pressure conditions (*n* = 33) concerning the associations between participants’ model fitting coefficients and their self-reported anxiety scores.

Model Type	Adjusted *R*^2^	*F*-Value	*p*-Value	STAI-S			STAI-T		
				β	*t*-Value	*p*-Value	β	*t*-Value	*p*-Value
Original	-	-	-	-	-	-	-	-	-
1-s delay	-	-	-	-	-	-	-	-	-
2-s delay	-	-	-	-	-	-	-	-	-
3-s delay	-	-	-	-	-	-	-	-	-
4-s delay	-	-	-	-	-	-	-	-	-
5-s delay	0.066	3.272	0.080	0.187	1.100	0.280	0.214	1.256	0.219
6-s delay	-	-	-	-	-	-	-	-	-
7-s delay	-	-	-	-	-	-	-	-	-
8-s delay	-	-	-	-	-	-	-	-	-
9-s delay	-	-	-	-	-	-	-	-	-
10-s delay	0.076	3.625	0.066	−0.039	0.229	0.821	−0.070	0.403	0.690

The coefficients (β) of STAI-S and STAI-T in nonsignificant models (*p*-values for regression models: >0.05) are values if included in regression models. STAI-S: Spielberger State–Trait Anxiety Inventory (State); STAI-T: Spielberger State–Trait Anxiety Inventory (Trait). All variables were excluded from the regression model, and the adjusted *R*^2^ was zero.

## Data Availability

The data in this study is available upon request from the corresponding author.
